# A guide to classify tattoo motives in Mexico as a tool to identify unknown bodies

**DOI:** 10.1007/s00414-022-02814-0

**Published:** 2022-04-04

**Authors:** F. Holz, G. G. Carrillo-Núñez, E. G. Martinez Peña, A. A. Rivera Martinez, I. G. de la Peña Jiménez, Ramon Bonilla Virgen, M. A. Verhoff, Christoph G. Birngruber

**Affiliations:** 1 Department of Legal Medicine, University Hospital Frankfurt, Goethe University, Kennedyallee 104, 60596 Frankfurt am Main, Hessen Germany; 2 Departamento de Morfología, Centro Universitario de Ciencias de la Salud, Universidad de Guadalajara, Guadalajara, Jalisco México; 3 Comité de Alteraciones en el Desarrollo Sexual, Hospital Civil de Guadalajara “Fray Antonio Alcalde”, Guadalajara, Jalisco México; 4 Instituto Jalisciense de Ciencias Forenses, Guadalajara, Jalisco México; 5 Servicio de Cirugía Medicina Legal, Antiguo Hospital Civil de Guadalajara, Guadalajara, Jalisco México

**Keywords:** Identification, Identifier, Body modification, Classification, DVI, Tattoo

## Abstract

**Justification:**

In Mexico, the number of unidentified bodies has been steadily rising for years. By now, more than 50,000 bodies are considered unidentified. Forensic laboratories that could perform comparative molecular genetic investigation are often overburdened and examinations can take months. Therefore, pragmatic approaches that can help to identify more unknown bodies must be sought. The increased use of distinctive physical features might be one, and the high rate of tattooed people in Mexico points towards a great potential of tattoos as a tool for identification. The prerequisite for a comparison of antemortem (missing persons) and postmortem (unknown bodies) data is an objective description of the particularities, e.g., of the tattoos. The aim of this study was to establish an objective classification for tattoo motives, taking into consideration local preferences.

**Methods:**

In the database of the medicolegal services of the Instituto Jaliscience de Ciencias Forenses (IJCF) in Guadalajara, postmortem data of 1000 tattooed bodies from 2019 were evaluated. According to sex and age, the tattooed body localization and the tattoo motives were categorized.

**Results:**

The 1000 tattooed deceased showed tattoos on 2342 body localizations. The motives were grouped and linked to the following 11 keywords (with decreasing frequency): letters/numbers, human, symbol (other), plant, symbol (religious), animal, object, fantasy/demon/comic, tribal/ornament/geometry, other, unrecognizable.

**Conclusion:**

Using the proposed classification, tattoo motives can be described objectively and classified in a practical way. If used for antemortem (missing persons) and postmortem (unknown bodies) documentation, motives can be searched and compared efficiently—helping to identify unknown bodies.

## Introduction

The principle of the identification process of unknown bodies in general is straightforward. It consists of a comparison of postmortem (PM) data, collected during the examination of a corpse, with antemortem (AM) data of missing persons, stored in databases or provided by investigating authorities, family members, or other sources. But even if the theory of identification appears simple at first glance, the process can be a challenging task for various reasons, especially if the corpses are numerous, mutilated, or dismembered, or if the quantity or quality of AM and PM data and their exchange between institutions are insufficient.

Interpol developed recommendations for Disaster Victim Identification (DVI) that discriminate between primary identifiers (odontology, fingerprints, DNA) and secondary identifiers (physical characteristics, e.g., scars, moles, tattoos) as appropriate methods to clarify the identity of an unknown body [12]. The chosen method of identification “should be scientifically sound, reliable, applicable under field conditions and capable of being implemented within a reasonable period of time” and is not limited to the use of primary identifiers [12].

## Background

### The current situation in Mexico

The National Commission of Human Rights (Comisión Nacional de los Derechos Humanos, CNDH) describes the current situation in Mexico as a “humanitarian crisis” [18]. Since 2006, when former president Calderón declared the beginning of the war on drugs, more than 50,000 corpses remained unidentified nationwide until the end of 2020 [8, 21]. Nobody knows how many of them are among the 80,517 people that have officially been reported missing since 2006 [19].

In light of this development, insisting on the exclusive use of primary identifiers in the Mexican context would be counterproductive: DNA analyses are expensive and time-consuming, taking up to several months to be finished. Furthermore, a working DNA database that would allow the matching of AM profiles of missing persons or family members and PM profiles of unidentified bodies does not exist yet. For dental status and fingerprints, AM data is often not available—either due to missing documentation (dental status) or due to data protection concerns (fingerprints). Moreover, it seems that the cooperation and the exchange of information between different institutions involved in the identification process could be improved [3, 4]. Therefore, secondary identifiers, especially tattoos, should be more frequently used to contribute to identify unknown bodies.

### Tattoos are pieces of art—a benefit or a problem?

Recently published results indicate the great importance of tattoos as a possible tool of identification in Mexico: The retrospective study conducted at the Instituto Jaliscience de Ciencias Forenses (IJCF) in Guadalajara, Jalisco, showed that a proportion of 45.8% of incoming bodies were tattooed (*n* = 2045, male: 46.8%, female: 38,5%; age range: 15–87 years), with the age group between 20 and 29 years having the highest percentage of tattooed bodies (male: 67.9%, female: 61.2%). In total, 69.0% of the tattooed male decedents and 53.6 % of the tattooed female bodies had tattoos on more than one body region [5].

The sheer frequency of a physical feature alone does not make tattoos a useful tool for identification. Its usefulness largely depends on the availability and quality of AM and PM data, which is essential for comparability and crucial for the identification process. As tattoos are pieces of art, and commonly connotated with a symbolic meaning, their description often is subjective. However, for the purpose of comparison and identification, the description should be as objective as possible to lower inter-observer bias and to take into account the occasionally only rough characterizations in AM data, e.g., given by family members or friends.

Proposals to classify tattoos have been developed in the past [1, 17, 25]. But until now, to the authors’ best knowledge, a practical approach that takes into account contemporary and common motives has not been implemented in routine casework in Mexico yet [1].

### Goal

The main goal of the study was to examine the type and frequency of tattoo motives of autopsied bodies in Jalisco, Mexico, to establish an objective and more practical classification of the most common motives. The classification is intended to be a pragmatic tool in the identification process of unknown bodies in Mexico as the increasing numbers of unidentified bodies means a major burden for the civil society and the forensic institutes.

## Material and methods

Using the PM database of the IJCF in Guadalajara, Jalisco, containing the data of all bodies that underwent autopsy, a retrospective study has been performed.

The written reports and photo documentations of all incoming complete bodies from the Metropolitan Area of Guadalajara (about five million inhabitants) starting January 1, 2019, were consecutively reviewed up to a total of 1000 tattooed deceased. Bodies, where skin was no longer present or assessable due to peri- or postmortem changes, were excluded.

Sex, age, tattooed body localization, and the motives of the tattoos were evaluated. Concerning the tattooed body localization, the following six regions were discriminated: head and neck, trunk, shoulders and upper arms, lower arms and hands, genitalia and buttocks, legs and feet. Tattoos on head and neck, the lower arms, and the hands were classified as “visible” [5]. Regarding the motives, eleven keywords/groups were created with respect to the most common ones: letters/numbers, symbol (religious), symbol (other), object, human, plant, animal, fantasy/demon/comic, tribal/ornament/geometry, other, unrecognizable (Table 1).

**Table 1:** Proposal for a practical classification of tattoo motives

Keywords	Examples of matching motives
Letters/numbers	Letters, initials, names, words, sentences, numbers, dates
Human	Portraits, human bodies or parts thereof, skulls, bones
Symbol (religious)	Cross, praying hands, rosary, angel, Jesus, Christian saints (e.g., Virgen de Guadalupe, San Judas Tadeo), Santa Muerte, symbols of other religions (e.g., Hinduism)
Symbol (other)	Infinity sign, star, yin yang, flag, three dots, tear drop, “Hecho en Mexico,” Playboy bunny
Plant	Flower, leaves (e.g., *cannabis*), cactus, tree
Animal	Mammals, birds, fish, arachnids, insects, or parts thereof
Object	Buildings or parts thereof, gun, knife, car, clock, playing cards, dice
Tribal/ornament/geometric	Tribal, twirls, ornament, geometric shapes (e.g., triangle, rectangle, circle)
Fantasy/demon/comic	Dragon, fairy, leprechaun, demon, demonic face, mask, clown, Super Mario, Mickey Mouse
Other	Cosmetic tattoo (e.g., eyebrows), sea, water, clouds, mountain, landscape
Not recognizable	Tattoo itself is visible, but a particular motive is not recognizable (e.g., due to fading)

## Results

Regarding all the bodies within the evaluation period, 46.6% of the male bodies and 37.6% of the female bodies had at least one tattoo.

**Fig. 1:**
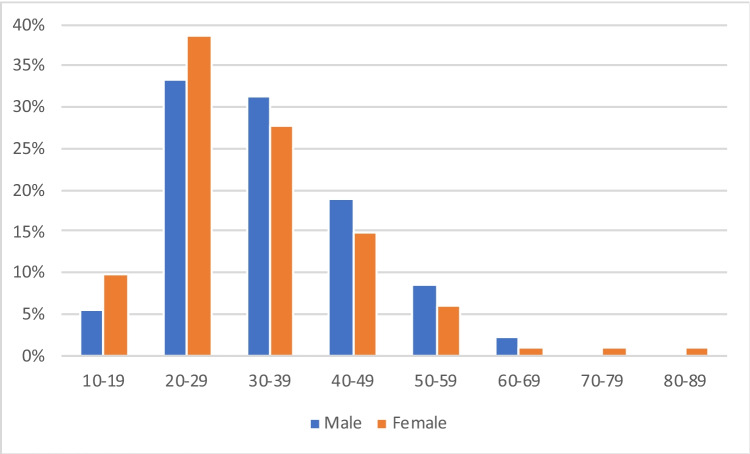
Age distribution of tattooed male and female bodies

Of the 1000 tattooed deceased, 89.9% were male (*n* = 899) and 10.1% female (*n* = 101). The median age of the tattooed deceased was 31 years, the mean age 34 years. The youngest tattooed body was that of a 15-year-old male, the oldest one that of an 87-year-old female (Fig. 1).

On each of the 1000 tattooed bodies, six regions were defined that were either classified as tattooed or not tattooed (see “Material and methods”), resulting in a total of 6000 body regions. The tattooed bodies presented tattoos on 2342 body regions. On average, males were tattooed on 2.4 body regions, females on 2.1 regions.

In total, 74.3% of the tattooed females and 63.7% of the tattooed males had tattoos on body locations that are likely to be exposed during everyday life (head, neck, forearms, hands).

**Fig. 2:**
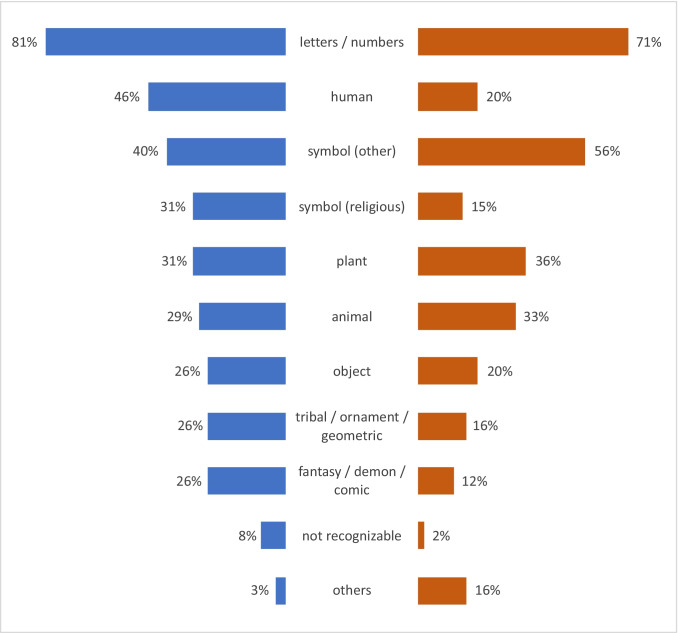
Frequency of tattoo motives in male (left) and female (right) bodies

Based on the type and frequency of the motives that were present on the bodies, a classification, based on keywords, was developed. Depending on the motive, several keywords could be assigned to a tattoo. The most common tattoo motives in male and female decedents contained numbers or letters, followed by pictorial representations of humans or body parts and symbols in males, and by symbols and illustrations of plants or parts thereof in females (Fig. 2).

**Fig. 3:**
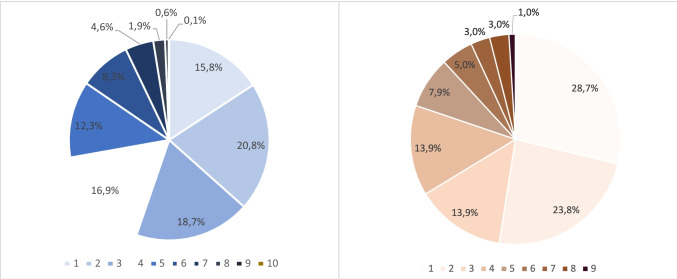
Percentage of tattooed bodies that showed a certain number of different tattoo motives (left: male, right: female)

To assess the diversity of tattoo motives, the number of different keywords that have been assigned to the tattoos on one body was recorded separately for male and female bodies (Fig. 3).

## Discussion

The identification of unknown deceased is a central task of forensic sciences and forensic medicine. Identification should be done out of respect for the deceased; it is in the interest of the rule of law and, above all, in the interest of the relatives. After all, certainty is the elementary prerequisite for mourning and processing traumatizing events [2]. Therefore, work should be done unremittingly to identify as many unknown bodies as possible. The identification method used in every individual case should be scientifically sound, and reliable, and yield results within a reasonable amount of time [12]. Its suitability strongly depends on the existing local conditions, particularities, and the population [6, 15, 22, 23, 26].

The numbers of unknown deceased in Mexico, which have been increasing since 2006, speak for themselves. The widespread fixation on DNA analysis alone should be reconsidered in view of overburdened laboratories and the lack of a nationwide DNA database of missing person and unknown bodies. Rather, it seems time for a pragmatic approach and alternatives: as a stand-alone identification method, as a complementary tool, or to plan and prioritize subsequent investigations, e.g., molecular genetic investigations [3–5].

The value of morphological methods to identify unknown bodies is beyond doubt [7, 9, 12]. To serve as an identifying feature, it needs to be unique and there must be a way to gain sufficient information of the feature on the missing person (AM), as well as on the unknown body (PM). In the best case, AM and PM data are stored in a standardized and accessible way that allows comparison in a timely manner to raise hints for an identity, or to confirm or exclude a suspected identity.

Tattoos reflect the identity of the tattooed person. They can be seen as storytellers of experiences gathered during life that can continue existing after death. On a simple view, tattoos are morphological features that are suitable to point out or ensure the identity of a living or deceased person [3, 8, 14].

Postmortem, tattoos are, as long as skin is present, usually easy to document during autopsy. On decomposed bodies, infrared photography and hydrogen-peroxide can be used as simple, quick, and affordable methods to gain proper information about their presence and motives [11, 20, 24]. Antemortem information is regularly generated during interviews with relatives by governmental or non-governmental institutions. For both, the antemortem side and the postmortem side, the information collected should be as accurate and objective as possible. Regarding this, problems can occur when tattoos are documented and described in order to compare them in databases. If databases only compare the text components of the descriptions, it can lead to both false-positive and false-negative identity suspicions. If, for example, the tattoo of a “rose” was reported in AM data by relatives as a “blossom” and PM documents the tattoo of a “flower” during the autopsy of the associated corpse, which was still unknown at the time of the autopsy, then an existing match can be overlooked if only the text is compared. The risk for errors becomes all the greater for more complicated or elaborate motives that may be interpreted differently by different viewers. Here, in the sense of an inter-observer error, different descriptions can easily occur with consequences for identification.

**Fig. 4:**
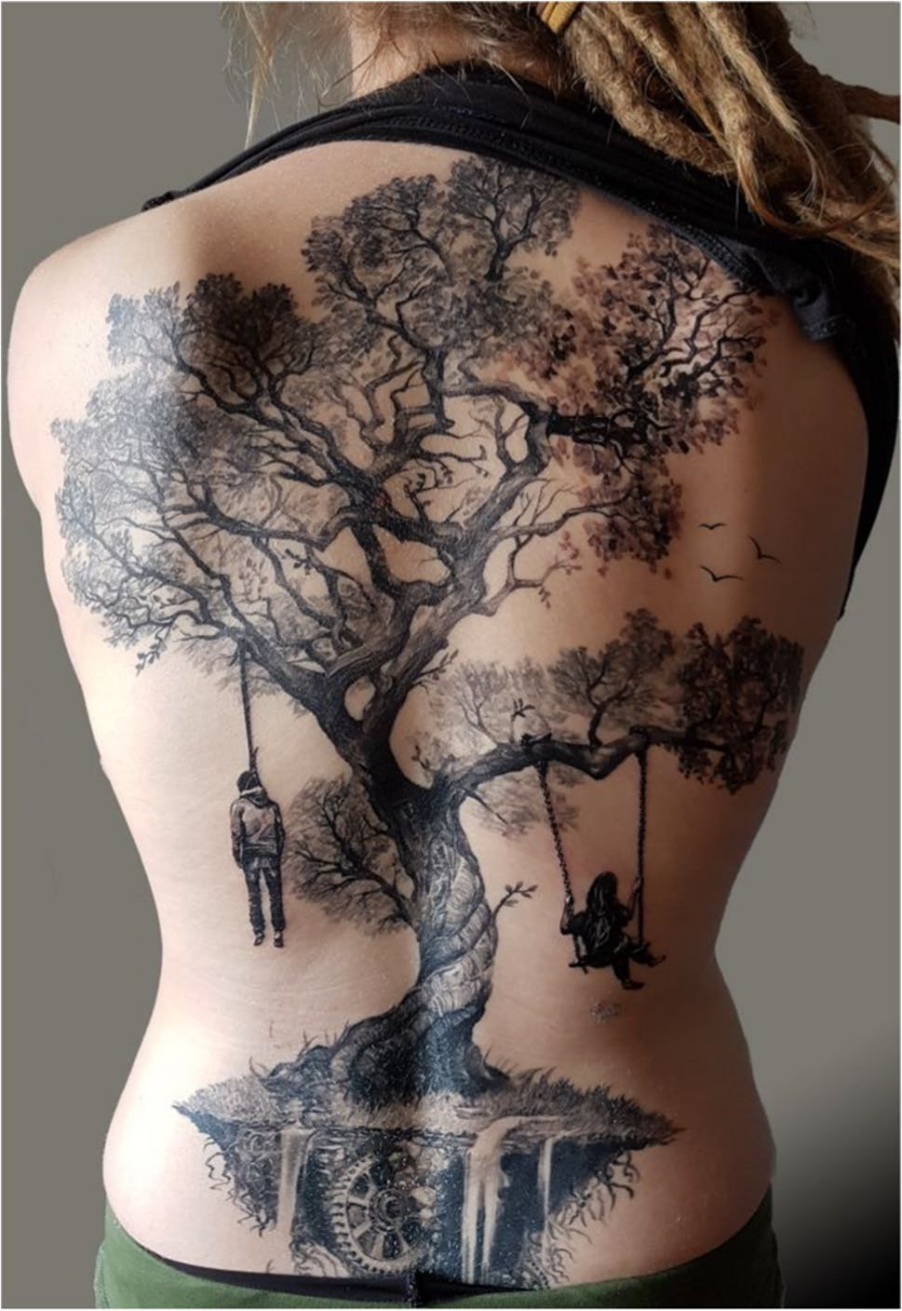
Example for the classification of a tattoo on the back of a living person: plant (tree)—human (hanging person and swinging person)—animal (birds)—object (wheel)—other (water)

In order to minimize interpretation errors, the authors developed a pragmatic and simple classification of tattoo motives. One or more basic keywords can be assigned to a tattoo to characterize it as objectively as possible (Fig. 4).

In the above-mentioned example, the tattooed “rose” as well as the AM-documented “blossom” and the PM-documented “flower” would be classified as “plant.” If the mere description is supplemented by keywords or a superordinate classification, the number of potential misses can be lowered.

Regarding keywords, in order not to lose information, it is suggested to use multiple keywords for motives that are difficult to assign to a single keyword. For example, an anchor should be classified as an “object” and at the same time as a “symbol,” the representation of a Virgen de Guadalupe—when viewed objectively—as “human” and “religious symbol.”

The suggested keywords are based on the most common tattoos of autopsied deceased in Jalisco. The authors are aware that frequencies and types of motives depend on zeitgeist, fashion, age, and gender of the tattooed, and other personal and regional characteristics. However, the selection of keywords developed and proposed on a collective from one Mexican state will be easily applicable to other national or international collectives, although frequencies may change.

Regarding a quick match, it should be noted that 81% of the examined tattooed male corpses and 71% of the examined tattooed female corpses had letters or numbers as a tattoo motive. This provides a relatively time-saving and simple opportunity for a text-based PM-AM or AM-PM search. Thus, a clue to an identity can be obtained with comparatively little effort and in a relatively short time, especially if the data matching between characteristics of unknown deceased and persons reported as missing is done digitally, continuously, and (semi-)automatically.

As a further step, the collection of AM and PM data should aim for comprehensive and detailed image documentation, or take into account available images of missing persons’ tattoos, which may come from relatives, investigative authorities, non-governmental organizations, and social networks. An automatic matching of tattoos with the help of a content-based image retrieval system like that proposed by Jain et al. is desirable, but not yet applicable in routine casework [10, 13, 14, 16]. Until a suitable technical system is developed and implemented, pragmatic solutions should be used to help identifying unknown bodies in a safe and quick way.

Considering the current situation in Mexico, the authors recommend an increased use of tattoos to help identifying unknown deceased persons. Depending on the circumstances of the individual case, they can be either used as a stand-alone or a complementary identification method, or to prioritize subsequent investigations. In any case, the proposed objective classification of tattoos will contribute to standardization and improved comparability of AM and PM data.
