# Correction to: A guide to classify tattoo motives in Mexico as a tool to identify unknown bodies

**DOI:** 10.1007/s00414-022-02827-9

**Published:** 2022-05-04

**Authors:** F. Holz, G. G. Núñez Carrillo, E. G. Martinez Peña, A. A. Rivera Martinez, I. G. de la Peña Jiménez, Ramon Bonilla Virgen, M. A. Verhoff, Christoph G. Birngruber

**Affiliations:** 1 Department of Legal Medicine, University Hospital Frankfurt, Goethe University, Kennedyallee 104, 60596 Frankfurt am Main, Frankfurt, Hessen Germany; 2 Departamento de Morfología, Centro Universitario de Ciencias de la Salud, Universidad de Guadalajara, Guadalajara, Jalisco México; 3 Comité de Alteraciones en el Desarrollo Sexual, Hospital Civil de Guadalajara “Fray Antonio Alcalde”, Guadalajara, Jalisco México; 4 Instituto Jalisciense de Ciencias Forenses, Guadalajara, Jalisco México; 5 Servicio de Cirugía Medicina Legal, Antiguo Hospital Civil de Guadalajara, Guadalajara, Jalisco México

**Correction to: International Journal of Legal Medicine (2022)**

10.1007/s00414-022-02814-0

The original online version of this article was revised due to an error in Figure 3b. The figure was replaced and now shows the missing colors.

The original article has been corrected.

