# Correction to: A guide to classify tattoo motives in Mexico as a tool to identify unknown bodies

**DOI:** 10.1007/s00414-022-02864-4

**Published:** 2022-07-06

**Authors:** F. Holz, G. G. Carrillo Núñez, E. G. Martinez Peña, A. A. Rivera Martinez, I. G. de la Peña Jiménez, Ramon Bonilla Virgen, M. A. Verhoff, Christoph G. Birngruber

**Affiliations:** 1 Department of Legal Medicine, University Hospital Frankfurt, Goethe University, Kennedyallee 104, 60596 Frankfurt am Main, Hessen Germany; 2 Departamento de Morfología, Centro Universitario de Ciencias de la Salud, Universidad de Guadalajara, Guadalajara, Jalisco México; 3 Comité de Alteraciones en el Desarrollo Sexual, Hospital Civil de Guadalajara “Fray Antonio Alcalde”, Guadalajara, Jalisco México; 4 Instituto Jalisciense de Ciencias Forenses, Guadalajara, Jalisco México; 5 Servicio de Cirugía Medicina Legal, Antiguo Hospital Civil de Guadalajara, Guadalajara, Jalisco México

**Correction to: International Journal of Legal Medicine (2022) 136:1105–1111**

**https://doi.org/10.1007/s00414-022-02814-0**

The online version contains an error in Author name. “G. G. Núñez Carrillo” should be changed to “G. G. Carrillo Núñez”.

The original article has been corrected.

